# Family Nurse Practitioners: an exploratory study ^*^

**DOI:** 10.1590/1980-220X-REEUSP-2022-0362en

**Published:** 2023-04-28

**Authors:** Marjorie Dantas Medeiros Melo, Isabelle Pereira da Silva, Luana Souza Freitas, Simone Karine da Costa Mesquita, Andrea Sonenberg, Isabelle Katherinne Fernandes Costa

**Affiliations:** 1 Universidade Federal do Rio Grande do Norte, Departamento de Enfermagem, Natal, RN, Brazil.; 2 Universidade Federal do Rio Grande do Norte, Programa de Pós-Graduação em Enfermagem, Natal, RN, Brazil.; 3 Pace University, School of Nursing, New York, United States of America.

**Keywords:** Advanced Practice Nursing, Nursing, Family Nurse Practitioners, Primary Health Care, Health Policy, Enfermería de Práctica Avanzada, Enfermería, Enfermeras de Familia, Atención Primaria de Salud, Política de Salud, Prática Avançada de Enfermagem, Enfermagem, Enfermeiras de Saúde da Família, Atenção Primária à Saúde, Política de Saúde

## Abstract

**Objective:**

To understand Family Nurse Practitioners’ practice, educational process and policy in the United States.

**Method:**

This is an exploratory, quantitative and qualitative study, developed in 2019 based on clinical observations and interviews with seven Family Nurse Practitioners in the state of New York. The interviews were transcribed and analyzed by the researcher through the observations made and also by the *Interface de R pour les Analyses Multidimensionnelles de Textes et de Questionnaires* software. The research was approved under Opinion IRB19-49.

**Results:**

Three content partitions emerged in the Descending Hierarchical Classification: 1) Being a nurse practitioner; 2) Educational paths and possibilities for action; and 3) Being political: a path to transformation. It was possible to describe skills and competencies considered outstanding in relation to other professional categories, points of improvement in their educational background and the importance of political representation and being active in role performance.

**Conclusion:**

This study highlights the similarities with the nurse working in Primary Care in Brazil and serves as a subsidy in the process of implementing this category in Brazilian Primary Care.

## RESUMO

**Objetivo:**

Compreender sobre a prática, processo educacional e política dos Enfermeiros de Prática Avançada da Família dos Estados Unidos.

**Método:**

Estudo exploratório, quanti-qualitativo, desenvolvido em 2019 a partir de observações clínicas e entrevistas com sete Enfermeiros de Prática Avançada da Família no estado de Nova Iorque. As entrevistas foram transcritas e analisadas pela pesquisadora mediante as observações realizadas e também pelo *software Interface de R pour les Analyses Multidimensionnelles de Textes et de Questionneires*. A pesquisa foi aprovada sob Parecer IRB19-49.

**Resultados:**

Três partições de conteúdo emergiram na Classificação Hierárquica Descendente: 1) O ser Enfermeiro de Prática Avançada; 2) Rotas Educacionais e possibilidades de atuação; e 3) Ser político: um caminho para transformação. Foi possível descrever competências e habilidades consideradas destaque em relação a outras categorias profissionais, pontos de melhorias na sua formação educacional e a importância da representação política e do ser ativo no desempenho da função.

**Conclusão:**

A realização deste estudo realça as similaridades com o enfermeiro atuante na Atenção Primária no Brasil e serve como subsídio no processo de implementação dessa categoria na Atenção Primária brasileira.

## RESUMEN

**Objetivo:**

Comprender la práctica, el proceso educativo y la política de las Enfermeras de Familia de Práctica Avanzada en los Estados Unidos.

**Método:**

Estudio exploratorio, cuantitativo y cualitativo, desarrollado en 2019 a partir de observaciones clínicas y entrevistas con siete enfermeras de práctica familiar avanzada en el estado de Nueva York. Las entrevistas fueron transcritas y analizadas por el investigador a través de las observaciones realizadas y también por el software Interface de R pour Analyses Multidimensionnelles de Textes et de Questionneires. La investigación fue aprobada bajo la Opinión IRB19-49.

**Resultados:**

Surgieron tres particiones de contenido en la Clasificación Jerárquica Descendente: 1) Ser Enfermero de Práctica Avanzada; 2) Rutas Educativas y posibilidades de acción; y 3) Ser político: un camino hacia la transformación. Fue posible describir competencias y habilidades consideradas sobresalientes en relación a otras categorías profesionales, puntos de mejora en su formación y la importancia de la representación política y ser activo en el desempeño de la función.

**Conclusión:**

La realización de este estudio destaca las similitudes con el enfermero que actúa en la Atención Primaria en Brasil y sirve como un subsidio en el proceso de implementación de esta categoría en la Atención Primaria brasileña.

## INTRODUCTION

Advanced Practice Nursing or Nurse Practitioner emerged in a context of changes in the epidemiological profile of the United States of America (USA), culminating in a need to adapt the health care model^(1)^.

An Advanced Practice Nurse (APN) is a registered nurse who has completed education to perform certain duties at an advanced level of care, determining their health needs through using critical thinking and advanced diagnostic judgment in order to create an individual and appropriate treatment plan for each situation, including prescribing medications and ordering laboratory tests^(2)^.

The vast majority of Nurse Practitioners (NP) in the USA are certified in the area of Primary Care and work in this scenario, given the range of possibilities that exist within their scope of action^(3)^. One of the possibilities for advanced practice nurses to work with is family health. Those who want to step up and treat the full range of patient populations, from infants to older adults, will become Family Nurse Practitioners (FNP)^(4)^.

FNP are care providers for families and communities, who work with autonomy and independence, and provide Primary Health Care (PHC) assistance, from diagnosis to treatment, focusing on health promotion and prevention actions^(4)^.

In 2022, the Pan American Health Organization (PAHO) addressed the development of Advanced Practice Nursing as a strategy to increase coverage and promote universal access, especially in PHC in Latin American and Caribbean countries. The main issues for investment in the nursing workforce were highlighted, driven by one of the worst pandemics faced^(5)^.

The global impact generated by the COVID-19 pandemic made 2020 the International Year of the Nurse and Midwife and 2021 the International Year of Health and Care Workers, highlighting the importance of these professionals in saving lives^(5)^. In view of this, the discussion of nurses’ autonomy as a professional capable of making decisions based on critical reflection of their actions in health for qualified assistance to populations was returned.

Nurses’ activities can identify people’s health conditions, based on their knowledge for investigation and with a critical view of the determinants and conditions of health situations^(2,4)^. APN can be the key to strengthening the PHC and the Unified Health System (SUS – *Sistema Único de Saúde*) by guaranteeing its basic principles. However, it is necessary to understand how advanced practice occurs in countries where it is already consolidated and to understand the difficulties, facilities and strategies to reflect on implementation in Brazil.

Considering the above scenario and in line with the global trend to expand the scope of nursing practice as a tool to help increase access to health, it is of great value to understand on-site, through clinical observations and interviews, how FNP’s practice occurs in the USA as a way to help the process of implementing this professional role in Brazil. Thus, the objective of the study was to understand about FNP’s practice, educational process and policy in the USA.

## METHODS

### Study Design and Outlining

This is exploratory quantitative and qualitative research, using the COREQ checklist.

### Data Collection Site

This is a study developed by a researcher with a sandwich PhD in progress, from September 2018 to September 2019, in New York, USA, at Pace University, on the Downtown and Pleasantville campuses, at the Pace University Health Clinic, in Montefiore Hospital’s worker care clinics and in an elementary school.

### Population and Participant Definition

The sample consisted of seven FNP. Respondents were enrolled using the snowball technique and data collection ended with data saturation.

### Selection Criteria

FNP and who had worked for at least 5 years were included. The purpose of job tenure as an inclusion criterion is to have a broad view of the role by professionals who have already been exercising it for a considerable time. Nurses on leave or who were not performing their duties were excluded.

### Instrument and Data Collection

**Chart 1.**
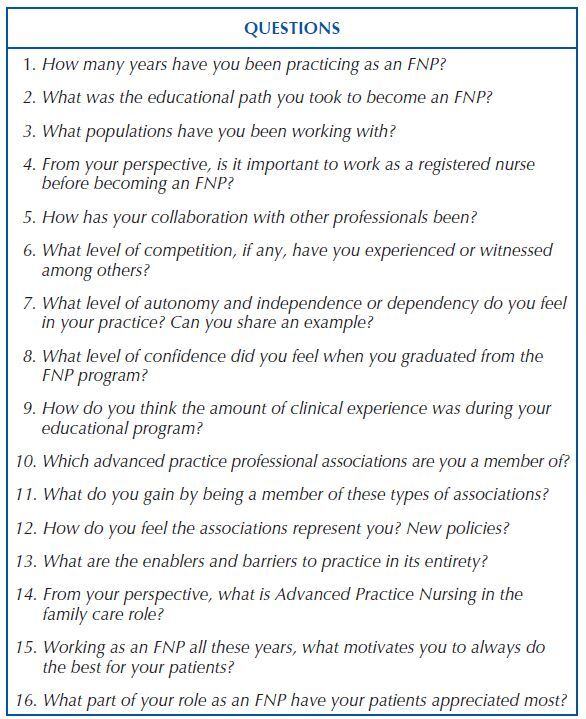
Interview questions – New York, NY, United States of America, 2020.

Source: own research.

Data collection was carried out through non-participant observation in clinics and hospitals in the state of NY, participant observation in classes of the FNP training course and through structured interviews composed of 16 questions, exposed in Chart 1, prepared by the author of the research, through reading and theoretical deepening arising from the observations made, with an average duration of 20 minutes each. Data were collected in order to describe a profile of the sample and observe how advanced nursing practice occurs, specifically in FNP, in order to understand these professionals’ role, how they are performed, what are their skills and competencies and what is professionals’ perceptions about of its role as FNP.

### Data Treatment and Analysis

Interview transcripts were sent to all research participants to verify content transcription accuracy and text meaning, with the researcher’s participant and non-participant observations being indispensable for composing, understanding and analyzing speech content. Data analysis was based on Max Horkheimer’s Critical Theory^(6)^. Upon approval of interviewees’ transcription, the transcribed textual content was submitted to lexicographical analysis, with the aid of the *Interface de R pour les Analyses Multidimensionnelles de Textes et de Questionnaires* (IRAMUTEQ) software. Descending Hierarchical Classification and similarity analysis were used as data treatment methods.

Thus, each text was characterized by the variables of interest (composition of the command line): time working as an FNP, sex and whether you had previous experience working as a registered nurse, for which a significance level of p ≤ 0.05 was considered. Criteria for inclusion of elements in their respective classes in the dendrogram were adopted: frequency greater than twice the average of occurrences in the corpus and association with the class determined by the chi-square value equal to or greater than 3.84 (p ≤ 0.05) and 95% significance.

### Ethical Aspects

To ensure participant anonymity, FNP were identified with the letters “FNP”, followed by the numbers 1, 2, 3, 4, 5, 6 and 7 (FNP1, FNP2, FNP3, FNP4, FNP5, FNP6, FNP7). After instructions on the research, participants signed the Informed Consent Form (ICF) and the authorization term for voice recording; then, data were collected in a private place.

In the USA, the researcher participated in the course on ethics, politics and research with humans offered by the Institutional Review Board (IRB), to receive mandatory certification with conditional assent to start the study, under number IRB19-49, in 2019, in which the stages of observation and interviews with nurses took place.

## RESULTS

Of the seven FNP, five were female (71.4%) and two were male (28.6%). Nurses had a minimum work experience as a FNP of 08 years and a maximum of 30 years, with the majority (71.4%) with operating time of 20 years or more. Regarding previous experience working as a registered nurse, it was identified that six had experience, after meeting the mandatory prerequisites for enrollment, and worked in different sectors: two at Pace University Clinical’s outpatient clinic; one at the clinical health clinic at the school to care for children and adolescents; two as professors in the FNP training course; and two at the occupational health clinic.

**Chart 2.**
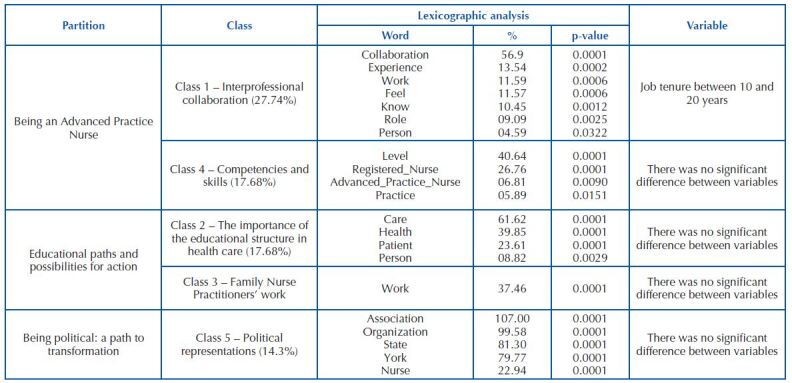
Dendrogram classes alluding to text corpus analysis – New York, NY, United States of America, 2020.

Source: own research. Caption: *p < 0.0001.

Corpus analysis from the interviews with FNP shows 15,480 occurrences of words, distributed in 1,660 forms. Through the Descending Hierarchical Classification, 427 text segments were analyzed, with retention of 76.81% of the corpus for constructing the five classes arising from content partitions, as shown in Chart 2.

The first partition, “Being an Advanced Practice Nurse”, resulting from text corpus analysis, provided an understanding of what it is to be an APN, from which two classes emerged: the importance of interprofessional collaboration in advanced practice (Class 1) and skills and competencies inherent to an APN and how time influences this process (Class 4). As for the vocabulary of Class 1, which makes up 27.74% of the quantitative analyzed, all the highlighted words were significant (p ≤ 0.05), showing that building collaborative relationships throughout their experiences enables exchange of knowledge, discussion of cases and mutual support in defining treatments and is of great value in FNP’s work, which can be highlighted in FNP4’s and FNP5’s speeches:

*(…) I have always been fortunate to work in an environment where you’re able to discuss cases, ‘to make sure you’re on the right track and doing the right thing for the patient. If I’m uncomfortable with something where I feel something is out of my scope, I always feel that I have backup, the collaborative physician, you know, checks my charts goes over it with me, shows me other things they may or may not have thought of, for that particular case. (FNP4)*

*(…) I’ve had very strong collaborative relationships with other practitioners. In my first role working in allergy and immunology that was such an unknown field to me, I knew so little about immunology and I worked with a really strong, very good immunologist, he was very willing to share knowledge and was very patient and worked with me and taught me continuing education courses, it was very positive*. *(FNP5)*

Class 1 showed statistical significance with the variable time working as an FNP in the range of 10 to 20 years of work, which shows that the construction of interprofessional relationships becomes more solid as trust is gained and communication skills grow up. FNP1’s speech highlights this:

*(…) I have found that building those relationships were very important but the most important component for building those collaborative relationships was the ability to communicate and to trust each other and understanding the role and the what that person brings to the table in terms of expertise. (FNP1)*

Class 4, arising from the first partition “Being an Advanced Practice Nurse”, mentions an FNP’s fundamental skills and competencies that differentiate them from other professions. FNP highlight points such as effective communication, differentiated view of primary health care and listening skills, which can be evidenced in FNP1’s and FNP5’s speeches:

*(…) I think in family practice, I think what we provide as advanced practice nurses is a unique understanding of primary care, a unique understanding of health maintenance and disease prevention and we understand that across the lifespan. (FNP1)*

*(…) but all the time we bring something unique to health care for our patients which is that we communicate with patients i think differently than the way that physicians are trained and we are very trained to listen to what a patient was telling you about their history. (FNP5)*

Regarding the specific composition of the Class 4 vocabulary, which makes up 17.68% of the analyzed quantity, the significant words evidenced were “level”, “Nurse Practitioner” and “Registered Nurse”, in order to emphasize the technical differences in the level of performance of APN and registered nurses, which are the ability to diagnose, prescribe medications and the appropriate treatment for a disease, which can be evidenced by FNP1’s, FNP2’s and FNP6’s speeches below:

*(…) the ability to diagnose and treat and prescribe and not necessarily the pharmacologic prescription but other prescription for on treatment so the responsibility then is increased and the level of clinical practice and how you think and how you problem solve are a lot different. (FNP1)*

*(…) the prescription practice is the biggest difference and being able to make a medical diagnosis as opposed to a nursing diagnosis. (FNP2)*

*(…) a RN can say this person has fever, like a technician person can say the temperature is 102. A nurse can say it’s fever and an RN can say it’s fever, and nurse practitioner can by doing a further evaluation, figure out, Okay, I think it’s an ear infection, or so they can take more of that data and analyze it further to a diagnosis and a treatment plan, and be able to prescribe and follow up and order tests, and, you know, make that diagnosis. (FNP6)*

The second partition, which included Classes 2 (17.68%) and 3 (16.16%), comprises the educational paths to becoming an FNP and their work environment, i.e., how the education process influenced their practice, if the number of hours in practice was satisfactory, points to be improved and what are the possibilities for FNP to act. Thus, initially analyzing Class 2, it is possible to observe in FNP2’s and FNP5’s speeches that the number of hours in practice were not enough for their training and transition to the field of practice:

*(…) I think it was about 600 hours that we had to complete and I don’t think it’s enough, especially when you’re doing family nurse practitioner, because it is so much knowledge that you have to know and you’re not going to be an expert at everything. (FNP2)*

*(…) I had 660 hours at that time of clinical experiences while I was in the program and I felt prepared but I knew that I needed to work in a setting where I would be supported, that I hadn’t learned everything that I needed to know. (FNP5)*

Still in Class 2, in line with what was presented above, the interviewees highlight as a point of improvement to complement the number of hours during the educational process. Residency is seen as a possible solution for FNP to gain confidence and autonomy after graduation, as can be demonstrated in FNP1’s, FNP3’s, FNP6’s and FNP7’s speeches.

*(…) What I do believe is that after the students finish with the initial 550 hours it would be wonderful to have these focus practice in a residency program. (FNP1)*

*(…) I think the nurse practitioner residency program, I think has a lot of potential to help young people or young providers in their not necessarily young an age but early in their experience to have that transition practice. (FNP3)*

*(…) why I think there’s a place for residency programs. In the NP world its relatively new, over the last 10 to 15 years, but and very little, it’s getting bigger now but I think that that would be very helpful. (FNP6)*

*(…) I think that a residency type program, after you graduate, whether it’s six months or a year, would really enhance your educational experience. (FNP7)*

Regarding Class 3, the word “work” is the only one to emerge, presenting statistical significance (p ≤ 0.05) and showing work environment characteristics, such as FNP and the satisfaction that work provides to them, as demonstrated by FNP1’s and FNP7’s speeches:

*(…) the most rewarding thing is probably when i worked in college health or i work in a private practice where i would get to know the patients and i would get to know their family members. (FNP7)*

*(…) we worked collaboratively and interprofessionally and we are now working towards policy change for overall patient care and population health within the United States so it is very exciting to see that happen. (FNP1)*

*(…) being able to have that open relationship and being able to have positive feedback and positive results from the either teaching or planning or medication the interventions that you do that really make this all worthwhile. (FNP7)*

Finally, Partition 3, “Being political: a path to transformation”, represented by Class 5, “Political representations” (14.3%), highlighted the importance of APN Associations and Organizations for expanding autonomy and gaining rights through changes in public policies in the country, FNP role in this struggle as a professional and what they receive from these political representations, which can be seen in FNP5’s and FNP6’s speeches below:

*(…) I gain from this associations continuing education meeting fellow providers and being part of a larger community professionally the NPA is very active in albany and passing legislation to make NP autonomous. (FNP5)*

*(…) I think we have to be involved in policy which were not enough and i wish i had been more active but i have not been i do not feel that we have a strong enough policy presence. (FNP6)*

All words contained in this class showed statistical significance, such as “association”, “organization”, “state”, “York” and “nurse’. This vocabulary denotes that APN’s political representations act as facilitators in guaranteeing the role of acting broadly and independently, as demonstrated in FNP3’s and FNP5’s speeches below:

*(…) I really feel that that state organization helps to advocate for nurse practitioners having the ability to work to the full scope of practice. So being a part of that state organization is important to me, because their work that they do helps to grow (…) so facilitators I think are our state national organizations to help us advocate for us having nurse practitioners who take on a leadership role who advocate as well. (FNP3)*

*(…) I think facilitators are professional organizations because they are the ones that are really assuring that we are able to practice at full scope as far as the legislators are concerned. (FNP5)*

**Figure 1.**
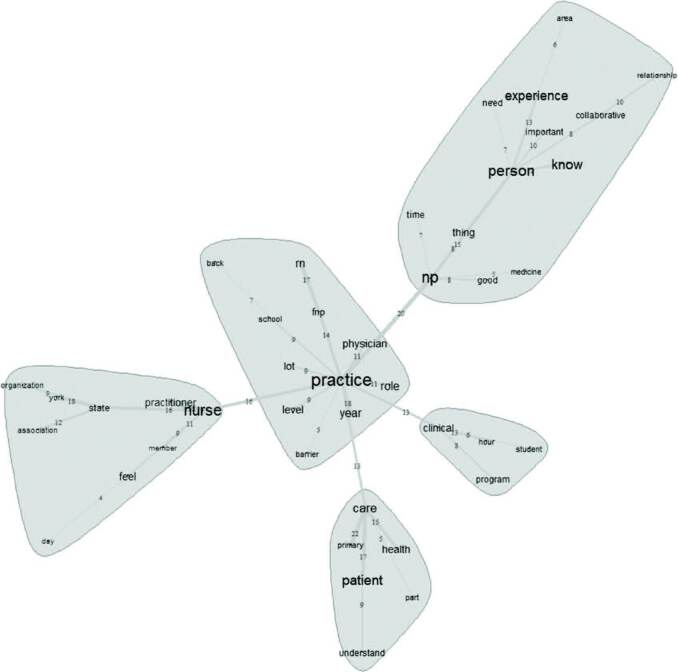
Similarity analysis of the text corpus, 2020.

Source: own research. np = nurse practitioner; fnp = family nurse practitioner; rn = registered nurse.

Figure 1 represents the similarity analysis, which summarizes the classified expressions referring to the understanding of FNP about the role.

According to Figure 1, in association with the expressions “practice”, “nurse” and “APN”, it denotes the differences in nurses’ and APN’s work. Therefore, the links with “care” and “clinical” represent the way in which care is provided within the practice environment of the interviewed nurses.

## DISCUSSION

The results presented aim to contribute to a better understanding of FNP role regarding their competencies and skills, training process and political importance of the role, with the aim of assisting in the process of implementing this category in the Brazilian PHC scenario.

The professionals participating in this study highlighted that they had collaborative relationships at work, based on respect and the mutual exchange of knowledge, where, whenever necessary, one assists the other in solving specific cases, without feelings of competition, with the aim of providing the best to their patients.

This fact highlighted in interviewees’ speeches was also noticed in clinical observations made in this study, where the harmonious and respectful interaction between physician and FNP was observed, for the discussion of more complex cases that required collaboration between professionals in defining the best treatment.

Supporting and strengthening relationships between nurses and physicians is recognized as beneficial in promoting a work environment that aims at high quality care. Having a collaborative health team requires professionals to respect, trust, share knowledge and also mutual responsibility for care, promoting better outcomes, decreasing workload, increasing job satisfaction and maintaining patient safety, and this patient-centered collaboration needs to be the fundamental foundation in the comprehensive care provided in all institutions, especially in PHC^(7,8,9)^.

Corroborating these statements, the study carried out with more than 500 professionals among APN and physicians in the State of New York, showed that the vast majority of professionals reported having a favorable teamwork. The same study also showed that teamwork between nursing professionals and physicians is an important predictor of their results. Those who work well in teams are more likely to report higher job satisfaction, lower intention to leave their job, and better quality of care^(10)^.

Another aspect highlighted by FNP are the skills and competencies characteristic of an APN that deserve to be highlighted and that differentiate them from other professions, such as advanced physical examination, critical clinical thinking and medication prescriptions.

It is important to emphasize that APN education must include clinical skills and competencies to enable effective clinical judgment as well as creative and low-cost solutions for the population. Knowledge based on concrete evidence generates skills in nurses, making them capable of facing emerging health problems and demands^(11,12,13,14)^.

At the international level, drug prescription is considered one of the main attributions of Advanced Practice Nursing, corroborating the findings of this study. Research carried out in Chile revealed positive evaluations regarding nurses’ prescriptive action such as better access to the service and care^(15)^.

In Brazil, medications are prescribed by the class representative entities and are aligned with the Ministry of Health for Primary Care protocols. With the advent of Advanced Practice Nursing and the consequent expansion of prescriptive autonomy through adequate training aimed at fulfilling this role, it would be possible to increase health service resolution and be an important element in the transformation of care, given the complexity of nursing and its transformative potential^(16,17)^.

It is important to highlight that the development of FNP’s competencies and skills is linked to their educational path, number of hours in clinical practices to gain confidence and better results in the transition from nurse to APN most respondents reported that the number of hours in training was not enough for their education, suggesting residency programs as a way to help the transition to the job market.

Accordingly, results of a study carried out with APN students demonstrate that these professionals feel the need for additional training that can improve direct clinical practice, with the main concern being safety related to medications. Moreover, factors such as years of previous clinical experience and additional training at a higher level were not significantly related to the improvement of clinical skills^(18)^. Thus, it appears the need for a complete training to strengthen the security of performance in multiple roles.

Furthermore, the importance of training APN is highlighted in order to promote the expansion of the scope of roles that nurses can play with autonomy in the search for greater resolution of health problems presented to their reality^(5)^.

A study carried out with APN who were in a residency program showed that newly graduated APN went from an initial state of “euphoria to shock and awe” in their first three months of practice to a final state of satisfaction at the end of residency through didactic and clinical support^(19)^.

In Brazil, Multidisciplinary Residency and Health Programs point out as one of the strong points in Advanced Practice Nursing implementation in the country. With an average duration of 24 months and a workload of 60 hours per week, they offer nurses the possibility of developing skills and competencies to act autonomously and confidently. In a study carried out with nursing residency and master’s students at *Escola de Enfermagem da Universidade de São Paulo* (EEUSP), the expanded clinical practice offered in these programs aimed at comprehensive care for individuals, families and communities, giving them a differentiated view of care, paying attention to each one’s singularities, is one of the pillars for Advanced Practice Nursing implementation in the country^(20)^.

To achieve APN status in the US, they need to take an educational path by taking the following steps: 1) Obtain a Registered Nurse (RN) diploma; 2) Pass the National Council Licensure Examination and receive a license to act as an RN; 3) Enroll in a Graduate Program (minimum master’s degree) and choose the target population they want to specialize in; and 4) Pass the Nurse Practitioner National Exam to obtain the license and act as an NP^(3)^.

Authors point out that from a meeting with Latin American countries, Brazil has strengths, but also difficulties to be overcome for Advanced Practice Nursing implementation in the country. Among the obstacles detected, there is the limited understanding of professional performance by national health agencies and the insufficient practical-clinical training offered by graduate programs^(21)^. The latter could benefit from minimal professional practice as a prerequisite for entering training programs.

As for regulatory issues, in line with the content of interviewees’ speeches, political representation appears as the last category and deserves to be highlighted, since, for developing the role of Advanced Practice Nursing, it is necessary professionals’ involvement with their representative entities in the fight for regulation of their activities and conquest of rights.

Political regulation is a major obstacle in Advanced Practice Nursing implementation, as there is still no consensus on this in countries that have already complied with Advanced Practice Nursing, and there is wide variation in educational requirements, regulation, limits and scope. The regulation aspect varies according to the jurisdiction of the country, and this situation can favor certain places or result in unnecessary limitations to Advanced Practice Nursing due to lack of clarity of roles and role conflicts, for instance^(22)^.

Regulation is fundamental to the identity, structure and type of services offered by a professional. How nursing is regulated can facilitate or impede its ability to remain relevant and to provide needed services^(22)^.

Nursing is constantly evolving and reaching positive levels on the world stage, providing high-quality care to the population with a holistic approach and care focused on the individuality of each patient, including health promotion, disease prevention, treatment and rehabilitation actions^(5,23)^.

In view of this, nurses’ political competency needs to be worked on more and more among professionals, for the profession to leave the role of mere spectator of the social transformations that happen around it, and have a critical stance capable of imposing oneself through praxis in situations that require positioning and union to achieve certain changes^(24,25)^.

The limitations of this study refer to the methodology characteristics, predominantly qualitative, which denotes a specific context so that generalization of data is not possible.

## CONCLUSIONS

The classes that emerged from this study could show a better view of FNP, given the researcher’s immersion in these professionals’ daily lives, being possible to observe their collaborative networks and way of acting in different clinical situations as well as to better understand their educational background through participation in classes for future FNP.

Advanced Practice Nursing with a focus on the family has unique characteristics that make it stand out within the team and reinforce the importance of valuing the role in primary health care within the USA scenario.

The in-depth understanding of this role provides subsidies for its implementation in Brazil, since understanding the necessary educational processes, which are the points of improvement during the training process and FNP core competencies and skills are valuable insights in establishing what the role is and how we can incorporate it into our setting.

It is necessary that nursing increasingly understands its place of action in the world as a sociopolitical and complex being capable of articulating knowledge and practice in search of its autonomy. It is necessary for the class to unite in favor of expansion of rights and understand that it can fly higher and higher in the path of conquest for a more autonomous and strengthened nursing.
